# Using the co-expression network of T cell-activation-related genes to assess the disease activity in Takayasu’s arteritis patients

**DOI:** 10.1186/s13075-021-02636-2

**Published:** 2021-12-16

**Authors:** Yixiao Tian, Jing Li, Xinping Tian, Xiaofeng Zeng

**Affiliations:** Department of Rheumatology and Clinical Immunology, Chinese Academy of Medical Sciences & Peking Union Medical College, National Clinical Research Center for Dermatologic and Immunologic Diseases (NCRC-DID), Ministry of Science & Technology, State Key Laboratory of Complex Severe and Rare Diseases, Peking Union Medical College Hospital (PUMCH), Key Laboratory of Rheumatology and Clinical Immunology, Ministry of Education, No.1 Shuai-Fu-yuan, Dongcheng District, Beijing, 100730 China

**Keywords:** Takayasu’s arteritis, Diagnosis, T cell activation, Disease activity, mRNA

## Abstract

**Background:**

There have been lacking reliable serum biomarkers in assessing the disease activity of Takayasu’s arteritis (TAK). This study aimed to assess the disease activity of TAK by assayed gene expression levels in peripheral mononuclear cells (PBMCs).

**Methods:**

The expression level of genes that essential in T cell activation in PBMCs in active TAK patients, inactive TAK patients, and healthy controls were detected by real-time fluorescence quantitative polymerase chain reaction, including TCR, CD28, CD40, CD40L, PD-1, PD-L1, PD-L2, CTLA4, TIGIT, TIM3, LAG3, CCL5, T-bet, RORC, and FOXP3. Gene co-expression network was established, and the signature of the topology structure in active TAK patients compared to the inactive TAK patients were extracted and described by formulas. Respectively, the disease activity was assessed by the routine serum biomarkers, including ESR, CRP, IL-6, and TNF-α, the gene expression level of TCR, CD28, T-bet, and RORC, as well as the signature of the topology structure, and the diagnostic efficacies were compared.

**Results:**

Compared with the inactive TAK patient group, the active TAK patient group had a greater clustering coefficient in the network consisting of genes that essential in T cell activation. When assessing the disease activity used this signature of topology structure, the sensitivity was 90.9%, the specificity was 100%, and the AUC was 0.98, which was greater than the AUCs of these biomarkers.

**Conclusions:**

The signature of the topology structure could distinguish the active TAK patients from inactive TAK patients. This maybe is a novel evaluation algorithm of disease activity.

**Supplementary Information:**

The online version contains supplementary material available at 10.1186/s13075-021-02636-2.

## Background

Takayasu’s arteritis (TAK) is a type of primarily large vasculitis. It is a chronic, relapsing, and progressive autoimmune disease, so it is very important to accurately assess disease activity during the follow-up. But there have been lacking validated composite measures of disease activity for TAK [1]. The disease activity markers for TAK the most widely used in the clinic are erythrocyte sedimentation rate (ESR), C-reactive protein (CRP), interleukin (IL-6), and tumor necrosis factor (TNF-α), but they are not very reliable. For instance, a study showed that ESR was normal in 28% of TAK patients diagnosed as active, while was elevated in 43% of TAK patients diagnosed in remission [2]. Besides, our prospective study which included 428 TAK patients showed that the sensitivity of ESR, CRP, IL-6, and TNF-α was 41.9%, 63.5%, 56.1%, and 48.2%, and the specificity was 88.1%, 88.7%, 85.8, and 60.3% [3].

In addition, there are other potential blood biomarkers for monitoring the progress of TAK [4], such as pentraxin-3 [5], YKL-40 [6], MMP-9 [7], MMP-3 [8], leptin [9], and serum amyloid A [9]. However, the diagnostic efficacies of these biomarkers have not been clinically validated. Recently, three studies indicate that serum complement is a potential biomarker for assessing the disease activity of TAK, including C3 [10], C1q [11], and C4a [12]. And C3 has a sensitivity of 69.9%, specificity of 66.7%, and an area under curves (AUC) of 0.715 [10], and C1q has a sensitivity of 77.8%, specificity of 64.9%, and AUC of 0.752 [11]. One study reported the serum chemokines were potential biomarkers for assessing disease activity of TAK, CCL2, CCL20, CXCL8, and CXCL10 had a sensitivity/specificity of 66.7%/67.2%, 54.2%/77.1%, 70.8%/72.1%, and 83.3%/54.1%, respectively [13]. From these studies, it seems to be difficult to find out a single blood biomarker with a reliable diagnostic efficacy.

In this study, we tried a new algorithm that using the topology structure of the gene co-expression network to assess the disease activity of TAK patients because we observed that there were many significant signatures in the gene co-expression network in active TAK patients differing from inactive ones. Furthermore, we compared the diagnostic efficacies of three methods, including the topology structure of the gene co-expression network, the serum biomarkers (ESR, CRP, IL-6, and TNF-α), and the mRNA level of gene (TCR, CD28, T-bet, and RORC) expression.

## Methods

### Patients

Treated TAK patients fulfilling the 1990 ACR criteria [14] were enrolled. And we assessed the disease activity of TAK by the 1994 NIH criteria [15], which included the following:

1. Systemic features, such as fever, musculoskeletal (no other cause identified)
2. Elevated ESR
3. New onset or aggravated features of vascular ischemia or inflammation, such as claudication, diminished or absent pulse, bruit, vascular pain (carotidynia), asymmetric blood pressure in either upper or lower limbs (or both)
4. Typical angiographic features

If a TAK patient had two or more features, he was defined as “active TAK patient”; otherwise, we diagnosed the patient was at remission stage, and the patient was defined as “inactive TAK patient”.

Written informed consent was obtained from all participants and the study was performed in accordance with the Declaration of Helsinki. And this study was approved by the Institutional Review Board of Peking Union Medical College Hospital, Beijing, China (S-478).

### Collection and processing of human blood samples

Peripheral blood mononuclear cells (PBMCs) were isolated from patients by density-gradient centrifugation. Total RNA was prepared from the PBMCs using Trizol reagent (15596026, Thermo Fisher Scientific) [16]. The RNA samples were diluted in RNase-free water, denatured at 65 °C for 10 min. RNA concentration and purity were determined spectrophotometrically, and the RNA integrity was verified by denaturing RNA gel electrophoresis.

### Real-time fluorescence quantitative polymerase chain reaction (RT-qPCR)

RNA was reverse transcribed using the PrimeScript™ RT reagent Kit with gDNA Eraser (RR047A, Takara). Genomic DNA (gDNA) was eliminated at 42 °C for 2 min. Reverse transcription was performed using the following conditions: 37 °C for 15 min, 85 °C for 5 s. RT-qPCR reactions were performed with the iTaqTM Universal SYBR® Green Supermix (725124, Bio-Rad), and primers were listed in Supplementary Table 1. The temperature cycle parameters in an Applied Biosystem 7900HT1 were as follows: 95 °C for 30 s and 40 cycles of 95 °C for 30 s, 56 °C for 30 s and 72 °C for 40 s followed by a hold at 72 °C for 40 s. Gene expression was calculated using the 2^−ΔΔCq^ method. Melting curve analysis was performed from 65 to 95 °C. The internal reference genes used for the comparison of HCs and TAK patients were B2M and SDHA. The internal reference genes used for relative RNA quantification only among TAK patients were YWHAZ and HPRT1.

### Establishment of the model for the assessment of the disease activity

**Fig. 1:**
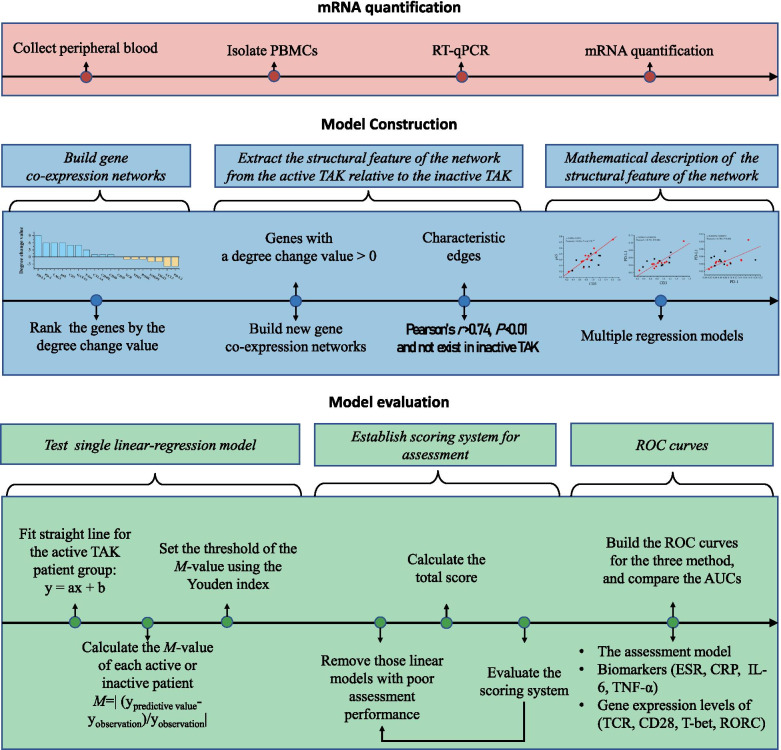
Modeling flow chart. PBMC, peripheral blood mononuclear cell. TAK, Takayasu’s arteritis. ROC, receiver operating characteristic curve. AUC, area under curve

After acquiring the expression data (∆Cq values), two gene co-expression networks were built for the active TAK group and the inactive TAK group, respectively. Then, characteristics of network structures were extracted from the active TAK group compared to the inactive TAK group. The characteristics of network structures were described by a group of linear regression equations. And the degree of each individual sample overfitting to the model was quantified. The model was constructed and assessed as Fig. 1 showed.

#### Build gene co-expression networks

Two gene co-expression networks were built based on gene expression correlation by the pairwise Pearson correlations between any two genes—one network from the active TAK patients, and the other from the inactive TAK patients. A *P* value of less than 0.05 was considered significant. Each node represented a gene, and each edge represented a correlation of expression level. Network analysis was performed using Cytoscape V.3.7.2.

#### Extract the structural feature of the networks

To extract the structural feature of the networks from the active TAK group relative to the inactive TAK group, in this study, a characteristic network structure was defied to meet the following two conditions:

1. Consisting of the genes with a degree change value of more than 0
2. Consisting of edges representing a correlation with a Pearson’s *r* greater than 0.74 and a *P* value less than 0.01 and only existing in the inactive TAK group or the active TAK group, which were defied as the characteristic edges

#### The degree change value of genes

The co-expression degree of a gene in the co-expression network was the number of edges the node has connecting to others. The degree change value = degree_active_- degree_inactive_.

In organisms, genes form molecular networks, these molecular networks tend to be modular, and similar modules combine to function [17, 18]. If there is a linear correlation between the expression levels of two genes, the two genes are likely closely related in function [19, 20]. Degree is one of most common topological measurements in network analysis. For example, the hub genes, which have a highest degree, are expected to play an important role in understanding the biological mechanism of response under stresses/conditions. Thus, genes with a high degree are generally considered to play an important role in maintaining the function of the network.

### Establish a mathematical model used to assess TAK disease activity based on the characteristic topological structure

The mathematical model consisted of all regression equations referring to each edge in the characteristic network structure. Next, we assessed the degree of each individual sample overfitting to the mode. To quantify how well a sample fit the model, two new parameters were introduced, including the *M value* and the *total score*. A threshold of the total score was set using the Youden index, and the patient that got a total score more than the threshold was diagnosed as being in the active TAK phase.

#### M value

To qualify how well the data of an individual sample fit a regression line, we calculated the relative error (RE) as the ratio of the absolute error (AE) and the observed value of gene expression level. The AE is the absolute value of the difference between the predictive value and the observed value.

*M* value was introduced which was equal to the RE:

$$M=\mid \left({y}_{\mathrm{predictive}\ \mathrm{value}}-{y}_{\mathrm{observation}}\right)/{y}_{\mathrm{observation}}\mid .$$

Qualified the data of each individual sample fit every regression line one by one. The *M* value of each active or inactive patient was calculated. Then, the patients were ranked by *M* value. The threshold was set using the Youden index and was adjusted in due course. A score of 1 was given if a patient had an *M* value less than the threshold, 0 if a patient had an *M* value greater than the threshold.

#### Total score

Total score of a patient was the sum of each score of *M* value. The threshold of the total score was set using the Youden index.

### Assessment for the disease activity of TAK using the serum biomarkers

To compare the assessment effects from the three methods for the disease activity of TAK, including the mathematical mode, the serum biomarkers, and the mRNA level of gene expression, we calculated the total scores and their thresholds following the same method described above. The reference values of ESR, hs-CRP, IL-6, and TNF-α were listed in Table 2. A total score of the biomarkers was also set: a score of 1 was given if a patient had a serum level greater than the reference, 0 if a patient had a serum level less than the reference. The total score of a patient was the sum of all biomarker scores.

### Assessment for the disease activity of TAK using the mRNA level of gene expression

Relative mRNA levels of the genes, including TCR, CD28, T-bet, and RORC, were calculated. The threshold was set using the Youden index. A score of 1 was given if a patient had an mRNA level greater than the threshold, 0 if a patient had an mRNA level less than the threshold. A total score of the gene expression level that was the sum of all biomarker scores was also set. The threshold of the total score was set using the Youden index too. The patients with missing values in the biomarkers were excluded from the analysis of this biomarker.

### Evaluation and comparison of the three types of assessment methods

The sensitivity, specificity, positive predictive value, and negative predictive value of each indicator were calculated. The receiver operating characteristic curves (ROCs) for each method were built, and the area under curves (AUCs) was compared.

### Statistical analysis

Normality was assessed with a Kolmogorov–Smirnov. Normally distributed continuous variables were provided as mean ± standard deviation and non-normally distributed continuous variables as median (interquartile). A chi-square test was used for reporting associations between two categorical variables. Differences of continuous variables between groups were analyzed by the Mann–Whitney test. The correlation between gene expression levels was represented by the Pearson correlation coefficient. Models were otherwise validated by examining standardized residuals for normal distribution. Statistical analysis was performed using IBM SPSS statistic V.23 (Armonk, New York, USA). A *P* value of less than 0.05 was considered significant.

## Results

### Patients

In total, 20 TAK patients that were being treated with immunosuppressants (9 inactive ones numbered #1 ~ #9, and 11 active TAK patients numbered #10 ~ #20) and 10 healthy controls (HCs) were included. These active patients manifested clinical features of fever (9.1%), arthralgia (36.4%), elevated ESR (27.3%), new onset or aggravated features of vascular ischemia or inflammation (such as diminished or absent pulse, bruit) (54.5%), and new occurrence of stenosis and vessel thickness of multiple large and medium-sized arteries (81.8%). The demographic data and clinical features of patients were listed in Table 1. There was one missing value in the hs-CRP, the IL-6, and the TNF-α each. Notably, 8 active TAK patients (72.7%) had a normal level of ESR (0 ~ 20 mm/h), 7 active TAK patients (70%) had a normal level of CRP (0 ~ 8.00 mg/L), 8 active TAK patients (80%) had a normal level of IL-6 (< 5.9 mg/L), and 8 active TAK patients (80%) had a normal level of TNF-α (< 8.1 mg/L). On the other hand, 1 inactive TAK patient (11.1%) had an increasing level of ESR, and 2 inactive TAK patients (22.2%) had an increasing level of TNF-α (Table 2).

**Table 1:** Demographic data and clinical features of patients with Takayasu’s arteritis

		Active (*n* = 11)	Inactive (*n* = 9)	*P* value
Age (year)		39.36 ± 7.89	39.44 ± 11.24	0.82
Gender (male/female)		1/10	0/9	0.35
Disease duration (month)		118.0 (13.0, 200.0)	35.56±26.66	0.10
ESR (mm/h)		16.82 ± 11.34	11.89 ± 4.88	0.33
hs-CRP (mg/L)		7.13±7.31	0.84 (0.26, 1.93)	0.02
Interleukin 6 (pg/mL)		4.27±2.65	2 (2.00, 2.70)	0.10
TNF-α (pg/mL)		6.85(5.50,8.38)	6.60 ± 2.24	0.66
Corticosteroid	Used/non-used	10/1	8/1	0.88
	Dose (mg/d)^a^	10 (10, 40)	10 (6.25, 29.5)	0.37

*ESR* erythrocyte sedimentation rate, *hs-CRP* hypersensitive C reactive protein, *TNF-α* tumor necrosis factor-α

^a^Corticosteroid dose had been converted into the dose of prednisone

**Table 2:** Assessment for the disease activity of TAK patients using the topology structure of the gene co-expression network, the serum biomarkers, and the gene expression level respectively

					**Topology structure of the gene co-expression network**			
Patient	Age (year)	Sex	Medication		Total score	CD3, p65	CD3, LAG3	CD3, PD-1
			Corticosteroid (mg/d))	Other immunosuppressant drugs				
						*y* = 0.420*x* + 0.0755	*y* = 0.00367*x* + 0.000253	*y* = 0.116*x*− 0.0166
						*r* = 0.926	*r* = 0.7823	*r* = 0.7494986
						*P* = 4.4137E−05	*P* = 0.0044	*P* = 0.0080
#1	32	F	24	MTX	4	1	0	1
#2	51	F	10	MMF, MTX, INH	1	0	0	0
#3	39	F	0	MMF, MTX	4	0	0	0
#4	34	F	5	HCQ, AZA	4	0	1	0
#5	49	F	7.5	CTX, MTX	7	1	0	1
#6	28	F	7.5	HCQ	5	0	0	0
#7	26	F	45	MMF, CTX	1	0	0	0
#8	59	F	35	MTX	3	0	0	0
#9	37	F	10	MMF, MTX	7	0	1	1
#10	36	F	40	MTX	12	1	1	1
#11	32	F	10	CTX, MTX	9	1	0	1
#12	50	F	15	LEF	9	1	0	1
#13	40	F	15	MMF, MTX	11	1	1	1
#14	25	F	10	MMF, MTX	10	1	1	0
#15	38	F	10	HCQ, AZA	9	0	1	0
#16	34	F	45	MMF, MTX	11	1	1	1
#17	39	F	44	CTX	11	1	1	1
#18	40	F	10	MMF, MTX	6	1	1	1
#19	49	F	10	MTX	12	1	1	1
#20	50	M	0	MMF	9	1	1	0
**Threshold**	/	/	/		8	0.211	0.211	0.430
**Sensitivity**	/	/	/		90.9%	90.9%	81.8%	72.7%
**Specificity**	/	/	/		100.0%	77.8%	77.8%	66.7%
**Positive predictive value**	/	/	/		100.0%	83.3%	81.8%	72.7%
**Negative predictive value**	/	/	/		90.0%	87.5%	77.8%	66.7%
			**Biomarker**				**Gene expression level**	
Patient	Total score	ESR (mm/h)	hs-CRP (mg/L)		IL-6 (pg/mL)	TNF-α (pg/mL)	Total score	TCR
		(ref. range, 0~20)	(ref. range, 0~8.00)		(ref. range, < 5.9)	(ref. range, < 8.1)		mRNA level
#1	1	5	1		4.4	8.8	0	0.143
#2	1	21	2.86		3	7.5	0	0.331
#3	0	11	2.92		2	6.4	0	0.380
#4	0	12	0.84		2	4	2	0.945
#5	0	9	0.17		2	6	0	0.280
#6	0	7	0.91		2	6.1	1	0.305
#7	1	17	0.31		2	11	0	0.346
#8	0	12	0.44		2	4	1	0.195
#9	0	13	0.21		2.4	5.6	0	0.240
#10	0	16	0.77		2	7.8	4	0.685
#11	1	33	5.4		5.8	6.1	2	0.348
#12	1	19	14.7		5.7	8	2	0.423
#13	2	38	7.8		9.3	7.6	3	0.407
#14	2	16	23.7		7.5	4	3	0.893
#15	–	12	3.64		–	–	4	0.735
#16	0	6	0.34		2	5.2	3	0.969
#17	–	23	–		2.1	5.9	3	0.976
#18	1	1	5.85		3.5	24.5	1	0.442
#19	1	5	0.55		2.8	9.5	4	0.555
#20	1	16	8.51		2	5.6	2	0.256
**Threshold**	0.5	20	8.00		5.9	8.1	1.5	0.393
**Sensitivity**	77.8%	27.3%	30.0%		20.0%	20.0%	90.9%	/
**Specificity**	66.7%	88.9%	100.0%		100.0%	77.8%	88.9%	/
**Positive predictive value**	70.0%	75.0%	100.0%		100.0%	50.0%	90.9%	/
**Negative predictive value**	75.0%	50.0%	56.3%		52.9%	46.7%	88.9%	/

	**Topology structure of the gene co-expression network**								
Patient	CD3, PD-L1	CTLA4, GATA3	PD-L1, GATA3	PD-L1, LAG3	PD-L1, p65	LAG3, p65	GATA3, CD40L	PD-1, p65	PD-1, PD-L1
	*y* = 0.0549*x* + 0.000255	*y* = 14.*429x* + 0.0101	*y* = 11.58*x* + 0.0649	*y* = 0.0674*x* + 0.000206	*y* = 6.26*x* + 0.140	*y* = 74.5*x* + 0.183	*y* = 0.424*x* + 0.0790	*y* = 2.20*x* + 0.254	*y* = 0.294*x* + 0.023
	*r* = 0.899	*r* = 0.902	*r* = 0.809	*r* = 0.879	*r* = 0.843	*r* = 0.769	*r* = 0.858	*r* = 0.750	*r* = 0.746
	*P* = 0.00017	*P* = 0.00014	*P* = 0.0026	*P* = 0.00037	*P* = 0.0011	*P* = 0.0057	*P* = 0.00072	*P* = 0.0078	*P* = 0.0084
#1	0	0	0	1	0	0	0	0	1
#2	0	0	0	0	0	0	0	1	0
#3	1	1	1	0	1	0	0	0	0
#4	1	0	1	0	0	0	0	0	1
#5	0	0	1	1	1	0	0	1	1
#6	0	1	1	1	0	0	1	0	1
#7	0	0	0	0	1	0	0	0	0
#8	1	0	0	0	0	1	0	1	0
#9	0	0	1	1	1	1	0	0	1
#10	1	1	1	1	1	1	1	1	1
#11	1	1	1	0	1	1	1	0	1
#12	1	1	1	0	1	0	1	1	1
#13	1	0	1	1	1	1	1	1	1
#14	1	1	1	1	1	1	1	0	1
#15	1	1	1	1	1	0	1	1	1
#16	1	1	1	1	1	1	1	0	1
#17	1	0	1	1	1	1	1	1	1
#18	0	0	1	0	0	0	1	0	1
#19	1	1	1	1	1	1	1	1	1
#20	1	0	1	1	1	1	0	1	1
**Threshold**	0.279	0.173	0.701	0.226	0.281	0.242	0.318	0.281	0.363
**Sensitivity**	90.9%	63.6%	100.0%	72.7%	90.9%	72.7%	90.9%	63.6%	100.0%
**Specificity**	66.7%	77.8%	44.4%	55.6%	55.6%	77.8%	88.9%	66.7%	44.4%
**Positive predictive value**	76.9%	77.8%	68.8%	66.7%	71.4%	80.0%	90.9%	70.0%	68.8%
**Negative predictive value**	85.7%	63.6%	100.0%	62.5%	88.3%	70.0%	88.9%	60.0%	100.0%
	**Gene expression level**								
		CD28		T-bet		RORC			
Patient	Score	mRNA level	Score	mRNA level	Score	mRNA level	Score		
#1	0	0.010	0	0.018	0	0.0007	0		
#2	0	0.017	0	0.047	0	0.0006	0		
#3	0	0.032	0	0.060	0	0.0022	0		
#4	1	0.036	1	0.056	0	0.0013	0		
#5	0	0.032	0	0.063	0	0.0020	0		
#6	0	0.020	0	0.095	1	0.0010	0		
#7	0	0.016	0	0.038	0	0.0017	0		
#8	0	0.021	0	0.089	1	0.0019	0		
#9	0	0.027	0	0.066	0	0.0023	0		
#10	1	0.062	1	0.073	1	0.0030	1		
#11	0	0.036	1	0.052	0	0.0028	1		
#12	1	0.031	0	0.089	1	0.0013	0		
#13	1	0.033	1	0.042	0	0.0037	1		
#14	1	0.074	1	0.042	0	0.0037	1		
#15	1	0.035	1	0.075	1	0.0073	1		
#16	1	0.051	1	0.040	0	0.0075	1		
#17	1	0.088	1	0.097	1	0.0019	0		
#18	1	0.029	0	0.028	0	0.0009	0		
#19	1	0.035	1	0.077	1	0.0043	1		
#20	0	0.034	1	0.065	0	0.0038	1		
**Threshold**	/	0.033	/	0.069	/	0.0025	/		
**Sensitivity**	81.8%	/	81.8%	/	45.50%	/	72.7%		
**Specificity**	88.9%	/	88.9%	/	77.8%	/	100%		
**Positive predictive value**	90.0%	/	90.0%	/	71.4%	/	100%		
**Negative predictive value**	80.0%	/	80.0%	/	53.8%	/	75%		

*M* male, *F* female, *ESR* erythrocyte sedimentation rate, *hs-CRP* hypersensitive- C reactive protein, *TNF-α* tumor necrosis factor-α

The corticosteroid dose had been converted into the dose of prednisone. *MTX* methotrexate, *MMF* mycophenolate mofetil, *INH* isonicotinyl hydrazide, *HCQ* hydroxychloroquine, *AZA* azathioprine, *CTX* cyclophosphamide, *LEF* leflunomide. #1~# 9, inactive TAK patient. #10~#20, active TAK patient. The refrence genes for linear-regression models were YWHAZ and HPRT1. The refrence genes for gene expression levels were B2M and SDHA

### Increased mRNA level of TCR, CD28, GATA3, RORC, and FOXP3 in active TAK patients compared with the inactive ones

**Fig. 2:**
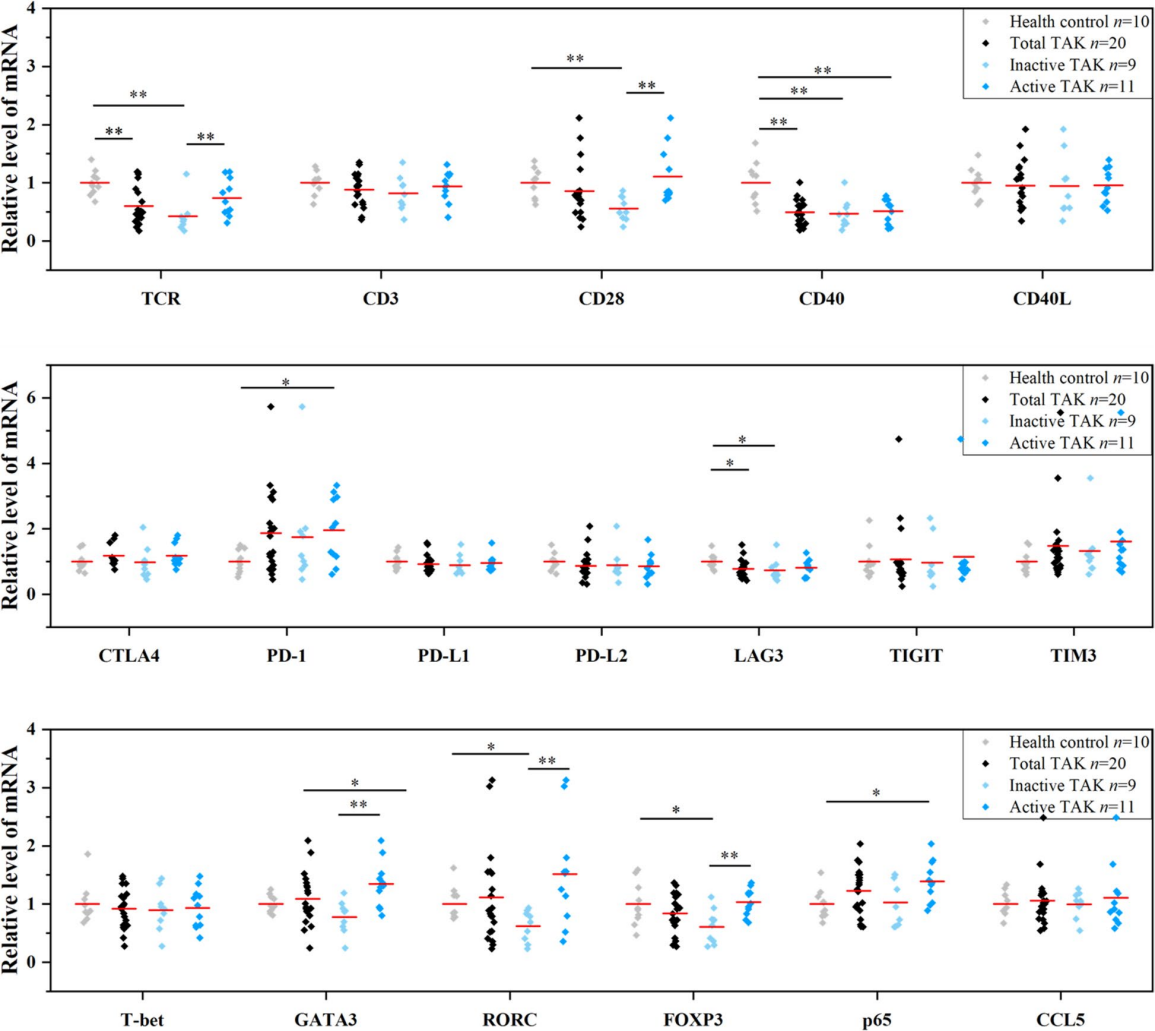
Relative mRNA expression levels of genes that essential in T cell activation in peripheral blood mononuclear cells in Takayasu’s arteritis (TAK) patients. Red lines indicate mean values. The reference genes were B2M and SDHA. Statistic was calculated with the Mann-Whitney *U* test. *P < 0.05. **P < 0.01. NS, no significant

Compared with the inactive TAK patients, the active TAK patients had an increased mRNA level of TCR, CD28, GATA3, and RORC in PBMCs. Besides, compared with the HCs, the active TAK patients had an increased mRNA level of PD-1, GATA3, and p65 in PBMCs. Notably, compared with the HCs, the TAK patients had a decreased mRNA level of TCR, CD40, and LAG3 in PBMCs, the inactive TAK patients had a decreased mRNA level of TCR, CD28, CD40, LAG3, RORC, and FOXP3 in PBMCs, and the active TAK patients had a decreased mRNA level of CD40. Interestingly, compared with the inactive TAK patients, the active TAK patients didn’t have a significantly increased mRNA level of T-bet and these immune checkpoint genes (Fig. 2).

### Structure features of gene co-expression network are described using linear formulas

**Fig. 3:**
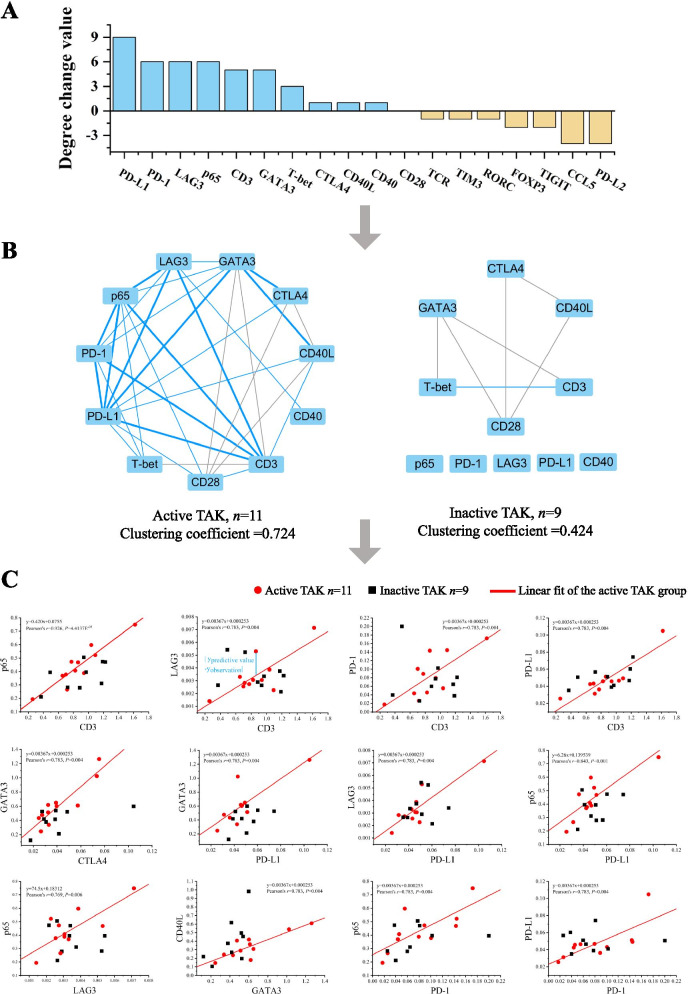
Analysis for the Topology structure of the gene co-expression network. RNA was extracted from PBMCs and quantified by RT-qPCR. The gene co-expression networks of inactive TAK and active TAK were constructed respectively. The Pearson correlation coefficient of any 2 gene expression levels was calculated to construct a correlation matrix. A *P* value of less than 0.05 was considered significant. The blue edge indicates the correlation only exists in the active TAK group or inactive TAK group. The bold line indicates the linear correlation that has a greater Pearson’s *r* than 0.74 and a smaller *P* value than 0.01. **A** Rank the genes by the degree change value (Degree_active_ − Degree_inactive_). **B** Exclude the genes with a decreasing degree, and construct a network map using the other genes. **C** The linear correlations between genes which were indicated by bold lines in **B**

Two gene co-expression networks were built for the active TAK group and the inactive TAK group, respectively (not shown), then the characteristics of network structures were extracted from the active TAK group compared with the inactive TAK group, and we established the assessment model using the algorithm shown in Fig. 1. The network analysis showed that compared with the inactive TAK group, the active TAK group had an increased degree of PD-L1, PD-1, LAG3, p65, CD3, GATA3, T-bet, CTLA4, CD40L, and CD40, while had a decreased degree of PD-L2, CCL5, TIGIT, FOXP3, RORC, TIM3, and TCR (Fig. 3A). The gene co-expression network consisting of the genes with a degree change value equal to or greater than 0 was built up, and the active TAK group characteristically had a greater clustering coefficient (0.724) than the inactive TAK group (0.424) (Fig. 3B). We defined the characteristic edge as the edge indicating the correlation with a Pearson’s *r* greater than 0.74 and a *P* value less than 0.01, and there were 12 characteristic edges in total. To make a mathematical description of the topology structure, the characteristic edges of the active group were described using a linear regression equation (Fig. 3C). To intuitively display the results, the points representing active patients or inactive patients were mapped to the same coordinate system. It could be seen from the figures that the red points which indicated the active TAK patients were more concentrated around the linear than the black points which indicated the inactive TAK patients (Fig. 3C).

### Compare the diagnostic efficacies of three methods to assess the disease activity using the topologic structure of the gene co-expression network, the serum biomarkers, and the gene expression levels

**Fig. 4:**
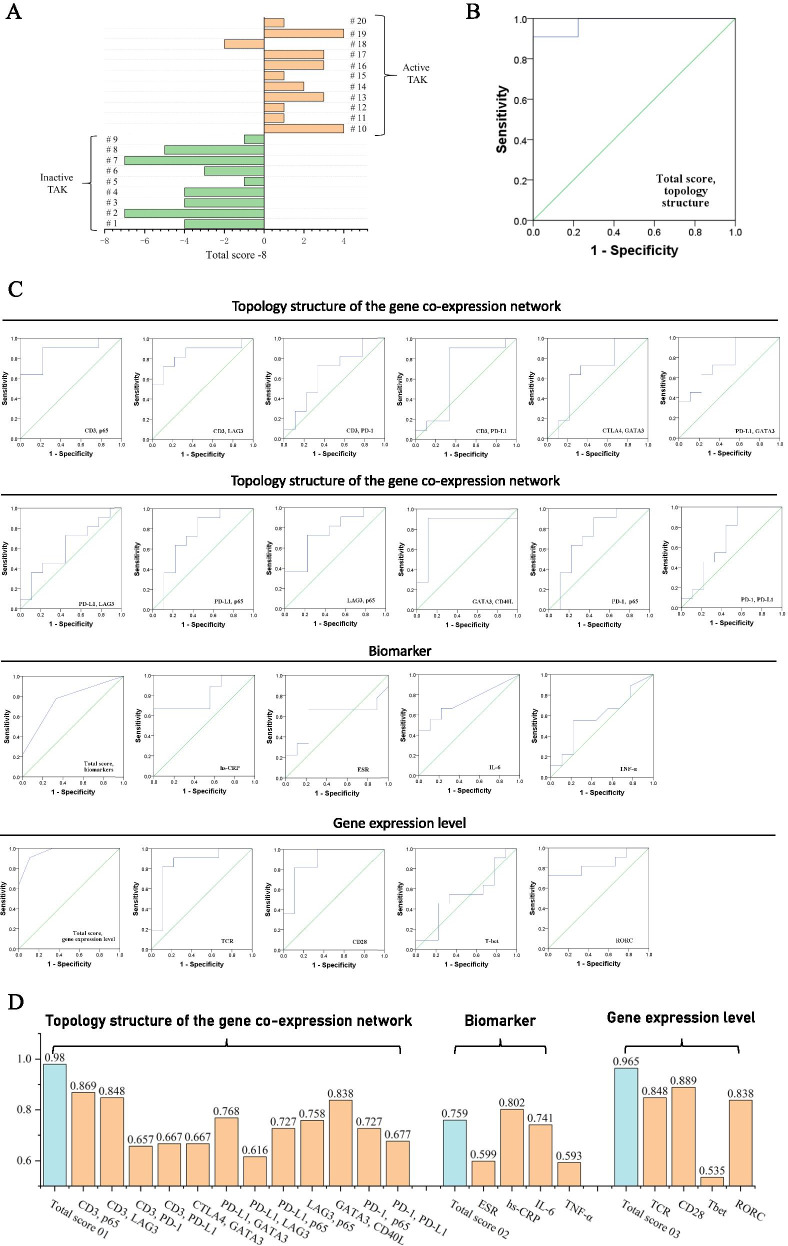
Assessment for the disease activity of TAK patients using the topology structure of the gene co-expression network, the serum biomarkers, and the gene expression level respectively. **A** Assessment using the scoring system established by the topology structure. The threshold of total score was 8. **B** The ROC of **A**. **C** The ROC of each indicator. **D** The AUC of each indicator. ROC, receiver operating characteristic curve. AUC, area under curve

We tested the three assessment methods and compared the diagnostic efficacies, and the evaluation details of each patient were listed in Table 2. When assessing the disease activity using the topologic structure of the gene co-expression network, the sensitivity, specificity, positive predictive value, and negative predictive value was 90.9%, 100%, 100%, and 90%, respectively (Table 2, Fig. 4A). The ROCs for each method were built and the AUCs were calculated (Fig. 4B, C), and the method adopting the topologic structure had the greatest AUC (Fig. 4D).

## Discussion

TAK is a chronic, relapsing, and progressive vasculitis, so it is very important to monitor the disease progression during the long-term follow-up. In this study, we presented a novel algorithm for assessing the disease activity of TAK using molecular diagnostics, which can reflect the T cell activation more directly, more sensitively, and more accurately than the serum biomarkers including ESR, CRP, IL-6, and TNF-α.

ESR, CRP, IL-6, and TNF-α are the commonly used indicators for assessing the disease activity of TAK in the clinic, but they all have a low sensitivity, which may contribute to missed diagnosis in some active TAK patients [1, 3]. Take CRP as an example. Most of the time, TAK is manifested by chronic inflammation instead of acute inflammation, which may lead to the normal levels of acute-phase proteins in peripheral blood. And TAK is sometimes mainly shown as local vascular inflammation, so it may be hard to find out inflammatory biomarkers with a high sensitivity correlating with the disease severity in peripheral blood. In addition, immunosuppression therapy may repress the increase of CRP level [21–23], which masks the inflammation activity. Therefore, the markers of acute inflammation might be not the best suitable indicator for assessing the disease activity of TAK, and the sensitivity often less than 80% might be inevitable [2, 3, 10, 11]

T cells contribute to the systemic and vascular manifestations of TAK [24–26]. The increase in the number of T helper cell 1(Th1) and Th17 cells in peripheral blood is correlated with TAK disease activity, and active TAK patients have more IFN-γ-, IL-6-, and IL-17A-producing T cells than inactive ones [24]. Besides, IFN-γ-, IL-6-, and IL-17A-producing T cells and T follicular helper cells exist in the vascular inflammatory infiltration in patients with TAK [24, 27]. In the active phase, a proportion of the autoreactive T cells in the arterial lesion come from the peripheral blood, so theoretically, the T cell activation can be detected in the peripheral blood, which might be more sensitive than the acute phase proteins.

In this study, we used three different methods to assess the disease activity, including the topology structure of gene co-expression network, the blood biomarkers, and the mRNA level of gene expression and compared the diagnostic efficacies of the three types of methods. As the results demonstrated, the diagnostic efficacies of the topology structures and the mRNA level of gene expression were better than the biomarkers. Notably, there is not only a topological indicator that can be used to assess disease activity, and we found that the Toll-like receptor-related gene groups were an indicator of the inactive TAK patients instead of active ones, which indicates that this algorithm could be used to quantify whether a certain signal pathway has been activated, and whether a certain an immune response has occurred in an individual patient instead of in a patient group. The topology structure of the gene co-expression network reflects the activation of signaling pathways in an individual patient, which may reveal more about the mechanism and contribute to the disease subtyping in autoimmune disease. However, the equations calculated by this study might need to be corrected with a large sample before they are applied to the clinic.

Additionally, our study provides several new findings on the disease activity of TAK at mRNA level. Firstly, the expression of GATA3 gene was increased in TAK patients presenting active disease compared to those in remission and to healthy controls, which suggested an ongoing Th2 response in the active stage of TAK, as GATA3 is a specific transcription factor of Th2 cells, which plays a critical role in the balance between Th1 and Th2 subsets in immune responses by promoting Th2 responses while inhibiting Th1 differentiation during the early stage of T cell differentiation [28, 29]. There are relatively fewer studies investigating the role of Th2 cells in the disease activity of TAK. Gao et al and Kong et al found the increased Th2 cell percentage in the peripheral blood in TAK patients compared to healthy controls [30, 31]. Studies have shown that the mRNA expression level of IL-4, which promotes the differentiation of Th2 cells while inhibits Th1 cells and is secreted by Th2 cells, was significantly higher in PBMCs of TAK patients than healthy controls, and the stimulated PBMCs of TAK patients, as compared with the controls, had higher mRNA gene expression of IL-4 [30, 32, 33]. Furthermore, the Th2 response promotes humoral immunity and antibody production [34]. Anti-endothelial cell antibodies are frequently presented in TAK patients, which may play a role in the pathogenesis [35]. However, it is generally believed that the inflammation of TAK is dominantly driven by T cells [36]. Although the role of B cells in the pathogenesis of TAK has been unclear, more and more evidence suggests that B cells and follicular helper T cells (Tfh) play an important role, including the disturbances of B cell homeostasis and increased Tfh cells in the peripheral blood, the tertiary lymphoid organs, B cells, and specific Tfh signature in areas of arterial inflammation [37–39]. Furthermore, we found that compared to the healthy controls, active-treated TAK patients have a higher level of GATA3, while inactive-treated TAK patients have a lower level of GATA3, which might account for the inconsistent results about whether there is an increased Th2 level in the peripheral blood in TAK patients. That is, some studies have detected an increased Th2 level but others have not, which might be related to not grouping TAK patients according to the disease activity [30, 31, 39]. Our findings provide new evidence at the mRNA level that Th2 cells and humoral immunity are involved in the pathogenesis of TAK.

Our study also showed that there were increased mRNA levels of TCR, CD28, RORC, and FOXP3 in active TAK patients compared to inactive TAK patients, which indicating that T cells and Th17 cells were involved in the inflammatory activity, which is consistent with previous findings [24, 40]. Interestingly, the mRNA levels of T-bet did not have a significant increase in active TAK patients compared to inactive TAK patients or healthy controls. Although some studies detected an increased level of Th1 cells in the peripheral blood of active TAK patients than that of inactive TAK patients [40], some studies did not detect an increase in Th1 cell level [30, 39], so it is controversial whether the level of Th1 cells in the peripheral blood of active TAK patients is elevated compared to inactive TAK patients. Besides, there were decreased mRNA levels of TCR, CD28, RORC, and FOXP3 in TAK patients in remission compared to healthy controls, which demonstrated an attenuated ability and a decrease number of T cells, and we speculate that which was related to the immunosuppressive therapy.

Secondly, we found that when comparing the active TAK patients to the inactive TAK patients, although there were increased mRNA levels of TCR, CD28, GATA3, RORC, and FOXP3, which indicate that T cells were involved in the disease activity of TAK, these mRNA levels of many immune checkpoints were not increased, such as CTLA4, PD-1, LAG3, TIGIT, and TIM3. We speculate that it might be related to the attenuated secretion of IL-10 in PBMCs of TAK patients [32, 41–43]. Notably, although these immune checkpoints were not increased in active stage than in inactive stage, the emerging gene co-expression revealed the activation of these immune checkpoints in active TAK stage, which suggests that gene co-expression network might more accurately reflect the activation of T cells than the gene expression levels in TAK.

This study also has some limitations. An important limitation is the lack of a new validation cohort of TAK patients to confirm the result. Second, TAK-naïve patients were not included in the analyses. The immunosuppressive and biologic therapy might suppress T cells and alter the parameters of the linear model. Last, the sample size was relatively small. However, the minor heterogeneity across samples and the clearly statistically significant group differences suggested the reliability of the results. As a next step, we plan to validate our model in an independent large randomized cohort, which includes four groups of TAK patients, such as inactive-naïve TAK patients, active-naïve TAK patients, inactive-treated TAK patients, and active-treated TAK patients.

## Conclusions

The activities of T cells in peripheral blood might reflect the disease activity of TAK more sensitively than some markers of acute inflammation, and the topology structure of the gene co-expression network of TAK patients is potential to be applied to assessing the disease activity of TAK in the clinic.

## Supplementary Information

**Additional file 1: Supplementary Table 1.** Primers used in real-time fluorescence quantitative polymerase chain reaction.

## Data Availability

Data and materials are available from corresponding author upon reasonable request.

## Abbreviations

TAK: Takayasu’s arteritis
PBMC: Peripheral blood mononuclear cell
Th: T helper cell
RT-qPCR: Real-time fluorescence quantitative polymerase chain reaction
HC: Healthy control
ROC: Receiver operating characteristic curve
AUC: Area under the curve
ESR: Erythrocyte sedimentation rate
CRP: C-reactive protein
IL-6: Interleukin-6
TNF-α: Tumor necrosis factor α
p65: RELA proto-oncogene NFκB subunit
PDCD1: Programmed cell death 1, also known as PD-1
PD-L2: Programmed cell death 1 ligand 2
CTLA4: Cytotoxic T lymphocyte associated protein 4
TIM3: T cell immunoglobulin domain and mucin domain 3, also known as HAVCR2, Hepatitis A virus cellular receptor 2
LAG3: Lymphocyte activating 3
TIGIT: T cell immunoreceptor with Ig and ITIM domains
TCR: T cell receptor
T-bet: T-box expressed in T cells, also known as TBX21, T-box transcription factor 21
GATA3: GATA-binding protein 3
RORC: RAR-related orphan receptor C
BCL6: BCL6 transcription repressor
FOXP3: Forkhead box P3
SDHA: Succinate dehydrogenase complex flavoprotein subunit A
B2M: Beta-2-microglobulin

## References

[1] Kim ESH, Beckman J (2018). Takayasu arteritis: challenges in diagnosis and management. Heart.

[2] Kerr GS, Hallahan CW, Giordano J, Leavitt RY, Fauci AS, Rottem M, Hoffman GS (1994). Takayasu arteritis. Ann Intern Med.

[3] Li J, Wang Y, Wang Y, Wang Y, Yang Y, Zhao J, Li M, Tian X, Zeng X (2020). Association between acute phase reactants, interleukin-6, tumor necrosis factor-α, and disease activity in Takayasu’s arteritis patients. Arthritis Res Ther.

[4] Tombetti E, Hysa E, Mason JC, Cimmino MA, Camellino D (2021). Blood biomarkers for monitoring and prognosis of large vessel vasculitides. Curr Rheumatol Rep.

[5] Tombetti E, Di Chio MC, Sartorelli S, Papa M, Salerno A, Bottazzi B, Bozzolo EP, Greco M, Rovere-Querini P, Baldissera E (2014). Systemic pentraxin-3 levels reflect vascular enhancement and progression in Takayasu arteritis. Arthritis Res Ther.

[6] Sun Y, Kong X, Wu S, Ma L, Yan Y, Lv P, Jiang L (2019). YKL-40 as a new biomarker of disease activity in Takayasu arteritis. Int J Cardiol.

[7] Sun Y, Ma L, Yan F, Liu H, Ding Y, Hou J, Jiang L (2012). MMP-9 and IL-6 are potential biomarkers for disease activity in Takayasu’s arteritis. Int J Cardiol.

[8] Tezuka D, Haraguchi G, Ishihara T, Takamura C, Suzuki JI, Isobe M (2012). The comparison in assessing disease activity among MMP-3, CRP and PTX3 according to max SUV from FDG-PET in Takayasu arteritis. J Cardiac Fail.

[9] Ma L, Yu W, Dai X, Yin M, Wang Y, Sun Y, Kong X, Cui X, Wu S, Ji Z (2019). Serum leptin, a potential predictor of long-term angiographic progression in Takayasu's arteritis. Int J Rheum Dis.

[10] Chen R, Ma L, Lv P, Lin J, Li C, Yan Y, Jin X, Dai X, Ji Z, Chen H (2021). Serum complement 3 is a potential biomarker for assessing disease activity in Takayasu arteritis. Arthritis Res Ther.

[11] Chen S, Luan H, He J, Wang Y, Zeng X, Li Y, Yuan H (2021). Serum C1q concentration is associated with disease activity in Chinese Takayasu arteritis patients: A case-control study. Health Sci Rep.

[12] Luo X, Zhang F, Huang Y, Wang Z, Wu Q. Plasma proteomic screening and validation of novel biomarkers in Takayasu’s arteritis. Clin Exp Rheumatol. 2021. Online ahead of print. 10.55563/clinexprheumatol/xv2o0c 33769262

[13] Dong H, Zhang Y, Zou Y, Chen Y, Yue J, Liu H, Jiang X (2021). Elevated chemokines concentration is associated with disease activity in Takayasu arteritis. Cytokine.

[14] Bloch DA, Michel BA, Hunder GG, McShane DJ, Arend WP, Calabrese LH, Edworthy SM, Fauci AS, Fries JF, Leavitt RY (1990). The American College of Rheumatology 1990 criteria for the classification of vasculitis. Patients and methods. Arthritis Rheum.

[15] Kerr G (1994). Takayasu's arteritis. Curr Opin Rheumatol.

[16] Rio DC, Ares M, Hannon GJ, Nilsen TW (2010). Purification of RNA using TRIzol (TRI reagent). Cold Spring Harb Protoc.

[17] Barabási AL, Oltvai ZN (2004). Network biology: understanding the cell's functional organization. Nat Rev Genet.

[18] Boccaletti S, Latora V, Moreno Y, Chavez M, Hwang DU (2006). Complex networks: structure and dynamics. Phys Rep Rev Sect Phys Lett.

[19] Lee HK, Hsu AK, Sajdak J, Qin J, Pavlidis P (2004). Coexpression analysis of human genes across many microarray data sets. Genome Res.

[20] Butte AJ, Tamayo P, Slonim D, Golub TR, Kohane IS (2000). Discovering functional relationships between RNA expression and chemotherapeutic susceptibility using relevance networks. Proc Natl Acad Sci U S A.

[21] Wang S, Ai J, Cui P, Zhu Y, Wu H, Zhang W (2020). Diagnostic value and clinical application of next-generation sequencing for infections in immunosuppressed patients with corticosteroid therapy. Ann Transl Med.

[22] Liu N, Liu JT, Ji YY, Lu PP (2011). Rosiglitazone regulates c-reactive protein-induced inflammatory responses via glucocorticoid receptor-mediated inhibition of p38 mitogen-activated protein kinase-toll-like receptor 4 signal pathway in vascular smooth muscle cells. J Cardiovasc Pharmacol.

[23] Müller B, Peri G, Doni A, Perruchoud AP, Landmann R, Pasqualini F, Mantovani A (2002). High circulating levels of the IL-1 type II decoy receptor in critically ill patients with sepsis: association of high decoy receptor levels with glucocorticoid administration. J Leukoc Biol.

[24] Saadoun D, Garrido M, Comarmond C, Desbois AC, Domont F, Savey L, Terrier B, Geri G, Rosenzwajg M, Klatzmann D (2015). Th1 and Th17 cytokines drive inflammation in Takayasu arteritis. Arthritis Rheumatol.

[25] Seko Y, Takahashi N, Tada Y, Yagita H, Okumura K, Nagai R (2000). Restricted usage of T-cell receptor Vgamma-Vdelta genes and expression of costimulatory molecules in Takayasu’s arteritis. Int J Cardiol.

[26] Seko Y, Sato O, Takagi A, Tada Y, Matsuo H, Yagita H, Okumura K, Yazaki Y (1996). Restricted usage of T-cell receptor Valpha-Vbeta genes in infiltrating cells in aortic tissue of patients with Takayasu's arteritis. Circulation.

[27] Desbois AC, Régnier P, Quiniou V, Lejoncour A, Maciejewski-Duval A, Comarmond C, et al. Specific follicular helper T cell signature in Takayasu arteritis. Arthritis Rheumatol 2021;73(7):1233–43. 10.1002/art.41672 33538119

[28] Zhang DH, Cohn L, Ray P, Bottomly K, Ray A (1997). Transcription factor GATA-3 is differentially expressed in murine Th1 and Th2 cells and controls Th2-specific expression of the interleukin-5 gene. J Biol Chem.

[29] Zhu JF, Yamane H, Cote-Sierra J, Guo LY, Paul WE (2006). GATA-3 promotes Th2 responses through three different mechanisms: induction of Th2 cytokine production, selective growth of Th2 cells and inhibition of Th1 cell-specific factors. Cell Research.

[30] Gao N, Cui W, Zhao LM, Li TT, Zhang JH, Pan LL (2020). Contribution of Th2-like Treg cells to the pathogenesis of Takayasu’s arteritis. Clin Exp Rheumatol.

[31] Kong X, Sun Y, Ma L, Chen H, Wei L, Wu W, Ji Z, Ma L, Zhang Z, Zhang Z (2016). The critical role of IL-6 in the pathogenesis of Takayasu arteritis. Clin Exp Rheumatol.

[32] Tripathy NK, Chauhan SK, Nityanand S (2004). Cytokine mRNA repertoire of peripheral blood mononuclear cells in Takayasu’s arteritis. Clin Exp Immunol.

[33] Pan LL, Du J, Gao N, Liao H, Wan J, Ci WP, Yang C, Wang T (2016). IL-9-producing Th9 cells may participate in pathogenesis of Takayasu’s arteritis. Clin Rheumatol.

[34] Mosmann TR, Coffman RL (1989). TH1 and TH2 cells: different patterns of lymphokine secretion lead to different functional properties. Annu Rev Immunol.

[35] Eichhorn J, Sima D, Thiele B, Lindschau C, Turowski A, Schmidt H, Schneider W, Haller H, Luft FC (1996). Anti-endothelial cell antibodies in Takayasu arteritis. Circulation.

[36] Watanabe R, Berry GJ, Liang DH, Goronzy JJ, Weyand CM (2020). Cellular signaling pathways in medium and large vessel vasculitis. Front Immunol.

[37] Hoyer BF, Mumtaz IM, Loddenkemper K, Bruns A, Sengler C, Hermann KG, Maza S, Keitzer R, Burmester GR, Buttgereit F (2012). Takayasu arteritis is characterised by disturbances of B cell homeostasis and responds to B cell depletion therapy with rituximab. Ann Rheum Dis.

[38] Clement M, Galy A, Bruneval P, Morvan M, Hyafil F, Benali K, Pasi N, Deschamps L, Pellenc Q, Papo T (2016). Tertiary lymphoid organs in Takayasu arteritis. Front Immunol.

[39] Matsumoto K, Suzuki K, Yoshimoto K, Seki N, Tsujimoto H, Chiba K, Takeuchi T (2019). Significant association between clinical characteristics and changes in peripheral immuno-phenotype in large vessel vasculitis. Arthritis Res Ther.

[40] Régnier P, Le Joncour A, Maciejewski-Duval A, Desbois AC, Comarmond C, Rosenzwajg M, Klatzmann D, Cacoub P, Saadoun D (2020). Targeting JAK/STAT pathway in Takayasu’s arteritis. Ann Rheum Dis.

[41] Gao Q, Lv N, Dang A, Li Z, Ye J, Zheng D (2019). Association of interleukin-6 and interleukin-10 expression, gene polymorphisms, and Takayasu arteritis in a Chinese Han population. Clin Rheumatol.

[42] Sawant DV, Yano H, Chikina M, Zhang Q, Liao M, Liu C, Callahan DJ, Sun Z, Sun T, Tabib T (2019). Adaptive plasticity of IL-10(+) and IL-35(+) T(reg) cells cooperatively promotes tumor T cell exhaustion. Nat Immunol.

[43] Bedke T, Muscate F, Soukou S, Gagliani N, Huber S (2019). Title: IL-10-producing T cells and their dual functions. Semin Immunol.

