# Supplementary material for: Using the co-expression network of T cell-activation-related genes to assess the disease activity in Takayasu’s arteritis patients
Source: Arthritis Res Ther. 2021 Dec 16;23:303. doi: 10.1186/s13075-021-02636-2 (PMC8675511; doi:10.1186/s13075-021-02636-2)
Supplement: Supplementary file 1 — Additional file 1: Supplementary Table 1. Primers used in real-time fluorescence quantitative polymerase chain reaction. [file 13075_2021_2636_MOESM1_ESM.pdf]

Supplementary Table 1 Primers used in real-time fluorescence quantitative polymerase chain reaction

Genes	NCBI Reference Sequence	Forward primer	Reverse primer
CCL5	NC_000017.11	CCAGCAGTCGTCTTTGTCAC	CTCTGGGTTGGCACACACTT
P65	NC_000011.10	GAAGAAGAGTCCTTTCAGCG	GGGATGACGTAAAGGGATAG
PDCD1	NC_000002.12	GTGCCTGTGTTCTCTGTGGA	TCCGCTAGGAAAGACAATGG
PD-L1	NC_000009.12	GGTTGTGGATCCAGTCACCT	TTGGTGGTGGTGGTCTTACC
PD-L2	NC_000009.12	ACCGTGAAAGAGCCACTTTG	GCGACCCCATAGATGATTATGC
CTLA4	NC_000002.12	CATGATGGGGAATGAGTTGACC	TCAGTCCTTGGATAGTGAGGTTC
TIM3	NC_000005.10	CTGCTGCTACTACTTACAAGGTC	GCAGGGCAGATAGGCATTCT
LAG3	NC_000012.12	GCGGGGACTTCTCGCTATG	GGCTCTGAGAGATCCTGGGG
TIGIT	NC_000003.12	ATACAAGAGCGAAGGTCTCACG	CTGAGTCTCCCATAACAGCGG
CD28	NC_000002.12	CTATTTCCCGGACCTTCTAAGCC	GCGGGGAGTCATGTTCATGTA
CD40	NC_000020.11	TTGGGGTCAAGCAGATTGCTA	GCAGATGACACATTGGAGAAGA
CD40L	NC_000023.11	ACATACAACCAAACTTCTCCCCG	GCAAAAAGTGCTGACCCAATCA
TCR	NC_000014.9	CCTTCAACAACAGCATTATTATTCCAG	CGAGGGAGCACAGGCTGTCTTA
CD3	NC_000001.11	GCCAGAACCAGCTCTATAAC	GGCCACGTCTCTTGTCCAA

T-bet	NC_000017.11	TGACCCAGATGATTGTGCTCCAGT	AATCTCGGCATTCTGGTAGGCAGT
GATA3	NC_000010.11	CACCACAACCACACTCTG	GCCTTCCTTCTTCATAGTCA
RORC	NC_000001.11	GTGGGGACAAGTCGTCTGG	AGTGCTGGCATCGGTTTCG
FOXP3	NC_000023.11	GTGGCCCGGATGTGAGAAG	GGAGCCCTTGTCGGATGATG
SDHA	NC_000005.10	CAGCATGTGTTACCAAGCTGT	GGTGTCGTAGAAATGCCACCT
HPRT1	NC_000023.11	TTTATTCCTCATGGACTAATTATGGA	CCTCCCATCTCCTTCATCAC
B2M	NC_000015.10	GAGGCTATCCAGCGTACTCCA	CGGCAGGCATACTCATCTTTT
YWHAZ	NC_000008.11	AGACGGAAGGTGCTGAGAAA	CGTTGGGGATCAAGAACTTT

CCL5, C-C motif chemokine ligand 5. *p65*, RELA proto-oncogene NFκB subunit. *PDCD1*, programmed cell death 1, also known as *PD-1*. *PD-L1*, *CD274*. *PD-L2*, programmed cell death 1 ligand 2. *CTLA4*, cytotoxic T-lymphocyte associated protein 4. *TIM3*, T cell immunoglobulin domain and mucin domain 3, also known as hepatitis A virus cellular receptor 2. *LAG3*, lymphocyte activating 3. *TIGIT*, T cell immunoreceptor with Ig and ITIM domains. *TCR*, T cell receptor. *T-bet*, T-box expressed in T cells, also known as T-box transcription factor 21. *GATA3*, GATA binding protein 3. *RORC*, RAR related orphan receptor C. *FOXP3*, forkhead box P3. *SDHA*, succinate dehydrogenase complex flavoprotein subunit A. *HPRT1*, hypoxanthine phosphoribosyl transferase 1. *YWHAZ*, yrosine 3-monooxygenase/tryptophan 5.
